# 75 Moving Together: Exploring Peer Relationships through Creative Dance in First Grade

**DOI:** 10.1093/eurpub/ckae114.175

**Published:** 2024-09-26

**Authors:** Angeliki Giatra, Maria Koutsouba, Nektarios Stavrou, Fotini Venetsanou

**Affiliations:** National and Kapodistrian University of Athens, Athens, Greece; National and Kapodistrian University of Athens, Athens, Greece; National and Kapodistrian University of Athens, Athens, Greece; National and Kapodistrian University of Athens, Athens, Greece

## Abstract

**Purpose:**

This study aimed to examine the effect of a creative dance (CD) program on first graders’ relationships with their peers. Peer relationships are an important component of a child’s self-concept, contributing positively to an emotionally supportive classroom climate, school adjustment, and the learning process. Additionally, they play a key role in a child’s socio-emotional health, particularly at transitional stages, such as entering primary school. CD is used as an educational tool to develop social-emotional skills and psychological well-being; however, no study has so far focused on its effects on the interpersonal relationships of first graders. This study will provide insight into how CD affects peer relationships at this developmental stage.

**Method:**

A total of 71 first-grade students participated and were randomly assigned to the intervention group (IG, n = 36) and the control group (CG, n = 35). The IG participated in a nine-week CD program conducted during the physical education (PE) classes, while the CG followed the national PE curriculum. The peer relationships subscale of the Pictorial Scale of Perceived Ability and Social Acceptance, Greek version, was administered to all participants through individual interviews, before and after the intervention; qualitative data were collected for IG through lesson observations by teachers (PE and classroom teacher), and whole class discussions.

**Results:**

The (gender*group) analysis of covariance applied to children’s post-test scores (covariate: baseline scores) showed significant differences between groups (F = 8.395, p< .005, η2=.11), with IG outperforming CG. Qualitative data revealed that CD helped children discover communication and cooperation strategies. The development of these skills led to a decrease in competitiveness and confrontation among peers. Whole-class discussions provided information about children’s perceptions of concepts such as cooperation, and respect for others and allowed them to become aware of their shared needs and expectations, and to reflect on their behavior, strengthening their friendship bonds.

**Conclusion:**

It can be concluded that CD cultivates first graders’ social-emotional skills related to peer relationships, and it affects their relationships positively. Based on the above, CD is proposed as an educational tool that can enhance children’s friendship bonds and reduce antisocial or aggressive behaviors that appear to be in raise nowadays.

